# Dynamic Preference for NADP/H Cofactor Binding/Release in *E. coli* YqhD Oxidoreductase

**DOI:** 10.3390/molecules26020270

**Published:** 2021-01-07

**Authors:** Rajni Verma, Jonathan M. Ellis, Katie R. Mitchell-Koch

**Affiliations:** 1 Department of Chemistry, McKinley Hall, Wichita State University, 1845 Fairmount, Wichita, KS 67260, USA; 2 Department of Chemistry, University of Wisconsin-Madison, 1101 University Avenue, Madison, WI 53706, USA; jmellis4@wisc.edu

**Keywords:** NAD(P)H-dependent oxidoreductase, zinc-containing alcohol dehydrogenase, cofactor binding and release, interdomain cleft dynamics, molecular dynamics simulations

## Abstract

YqhD, an *E. coli* alcohol/aldehyde oxidoreductase, is an enzyme able to produce valuable bio-renewable fuels and fine chemicals from a broad range of starting materials. Herein, we report the first computational solution-phase structure-dynamics analysis of YqhD, shedding light on the effect of oxidized and reduced NADP/H cofactor binding on the conformational dynamics of the biocatalyst using molecular dynamics (MD) simulations. The cofactor oxidation states mainly influence the interdomain cleft region conformations of the YqhD monomers, involved in intricate cofactor binding and release. The ensemble of NADPH-bound monomers has a narrower average interdomain space resulting in more hydrogen bonds and rigid cofactor binding. NADP-bound YqhD fluctuates between open and closed conformations, while it was observed that NADPH-bound YqhD had slower opening/closing dynamics of the cofactor-binding cleft. In the light of enzyme kinetics and structural data, simulation findings have led us to postulate that the frequently sampled open conformation of the cofactor binding cleft with NADP leads to the more facile release of NADP while increased closed conformation sampling during NADPH binding enhances cofactor binding affinity and the aldehyde reductase activity of the enzyme.

## 1. Introduction

YqhD is a homo-dimeric, NADP(H)-dependent *E. coli* oxidoreductase identified in 2003 [1]. Since its discovery, it has received considerable attention for bioengineering efforts [2], being targeted for its utility in biomass conversion to biofuels [3] and chemical feedstocks [4]. The enzyme was initially identified during the *E. coli* structural genomics program and was shown to be widely distributed throughout the bacteria kingdom [5]. YqhD enzyme works as a homodimer with Zn^2+^ in the active site, catalyzing the interconversion of alcohols and aldehydes with NADP and NADPH, respectively, as a cofactor. Although YqhD binds Zn^2+^, it has structural similarities to the iron-dependent (group III) alcohol dehydrogenase enzymes [6]. Initially, YqhD was proposed as NADP-dependent alcohol dehydrogenase with a weak affinity toward short- and medium- chain alcohols [7]. Later studies characterized this enzyme as a NADPH-dependent aldehyde reductase with a broad range of substrates, such as 3-hydroxypropionaldehyde [8], propanaldehyde, isobutylaldehyde [3], acrolein, and malondialdehyde [2,9]. The enzyme’s biological role was also evident in the reduction of various reactive aldehydes derived from membrane lipid peroxidation [9] and mitigation of furfural toxicity [10,11,12]. Other biosynthetic efforts with YqhD have involved its utilization in the production of aromatic alcohols [13], ethylene glycol, 1-butanol [14], 1,4-butanediol, acetol [15,16], 1,2-propanediol [17] and, in a pathway using CO_2_, 1-butanol [18].

Just a few years after YqhD’s characterization, it became a candidate for protein engineering studies toward the biological production of useful chemicals and fuels, including valorization of biomass-derived precursors. Protein engineering approaches have produced variants of YqhD with improved affinity and higher catalytic efficiency toward 3-hydroxypicolinic acid for the production of 1,3-propanediol [19]. Engineered strains for the production of 1,3-propanediol from d-glucose [20] and glycerol [21,22] have also utilized YqhD. The broad enzyme substrate scope, combined with this report on the dependence of enzyme conformational dynamics on cofactor oxidation state, makes YqhD useful as a model system to better understand NADP-dependent zinc bound oxidoreductases [23,24,25,26]. Despite the abundance of structural and kinetics data for the YqhD oxidoreductase, there are no reports (experimental or simulations) until now on the structure and dynamics of this enzyme in solution.

**Figure 1:**
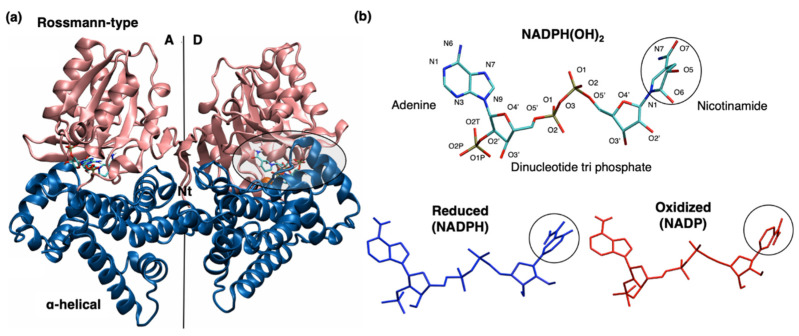
(**a**) YqhD dimer (comprised of monomers A and D) is in cartoon representation with the α-helical and Rossmann-type domains colored in blue and pink, respectively. The shaded region shows the cofactor-binding site of monomer D. Zn^2+^ is located at the interdomain face within the cofactor-binding site and is shown in orange colored VDW representation. The NADP cofactor is in licorice representation colored by elements (nitrogen, oxygen, hydrogen, carbon and, phosphorus in blue, red, white, carbon, and tan color, respectively). (**b**) NADP cofactor is present in the crystal structure as NADPH(OH)_2_ (with modification at C5 and C6 position in the nicotinamide moiety colored by elements and N and O atomic labeling) and in the MD simulations as NADPH and NADP in blue and red color, respectively. The black ovals highlight the nicotinamide moiety.

Herein, we report results from a set of molecular dynamics (MD) simulations of YqhD enzyme with both oxidized and reduced cofactors (NADP and NADPH) in aqueous solution. The study aims to rationalize the enzyme preference toward NADPH over NADP as a cofactor. The crystal structure [7] (PDB ID: 1OJ7) is the starting point for our simulations. In the crystal structure of the YqhD holoenzyme, the bound NADP cofactor is modified to NADPH(OH)_2_ due to oxidative stress during the crystallization process [7]. Figure 1 shows the dimeric structure of YqhD, which crystallizes as a tetramer in the asymmetric unit, as well as geometries of the modified and native cofactors (NADPH(OH)_2_, NADP, and NADPH). YqhD shows a lower Michaelis constant for NADPH [11] (K_M_ = 0.008 mM) than NADP [27] (K_M_ = 0.15 mM) and, concomitant with cofactor reactivity, a lower Michaelis constant for 1-butyaldehyde [9] (K_M_ = 0.67 mM) compared to 1-butanol [7] (K_M_ = 36 mM). Our simulation work of the YqhD homodimer has found that the cofactor oxidation state has a profound effect on enzyme structure and dynamics, which is consistent with differences in enzyme efficacy toward alcohols versus aldehydes. 

It has been observed previously that differences in cofactor oxidation states induce protein conformational changes [25,28] and also influence the cofactor binding affinities [29]. Along these lines, structural differences in cofactor binding for FucO oxidoreductase, a NADH-dependent enzyme with similarities to YqhD, were recently reported [30], while conformational changes in cofactor were shown to be critical in the pathway toward the transition state for horse liver alcohol dehydrogenase [31]. Recent work on product release, a rate-limiting step in dihydrofolate reductase (DHFR), indicates that NADPH induces product release through steric repulsion during conformational sampling of the closed excited state [32]. There is some variation in conformational dynamics (rates between exchange of states) when the DHFR-product complex is bound to oxidized (NADP) vs. reduced (NADPH) cofactor, but conformational exchange rates measured by NMR are on the same order of magnitude (1890 ± 80 s^−1^ bound to NADPH vs. 1420 ± 70 s^−1^ when bound to NADP^+^). 

The present work has gone beyond structural effects to computationally study how dynamics are affected by cofactor oxidation/reduction. To gain insight into dynamical preference of the YqhD protein towards NADPH over NADP cofactor, the article is organized as follows: the details of MD simulations are reported in the Methods section; the results section has the analysis of MD trajectories, with the main focus on structural and dynamical properties of YqhD enzyme and NADP/H cofactor binding; next, we discuss our results, focusing on the effect of cofactor oxidation/reduction on structure-dynamics and activity of the enzyme.

## 2. Results

### 2.1. Structural and Dynamical Properties of YqhD Enzyme

As mentioned in the Introduction section, the crystal structure has a modified NADPH(OH)_2_ cofactor, but in this work, simulations were performed for the YqhD homodimer with oxidized NADP and reduced NADPH cofactors (separately) for comparison (see Figure 1 for details). First, we performed an equilibration step involving 20 ns MD simulation in isothermal-isobaric (NPT) ensemble with each state. During the equilibration step, the YqhD protein adopts a conformation suitable for the binding of NADP and NADPH cofactors. Starting with equilibrated structures, we performed five 200 ns long independent MD simulations for each state, starting with different initial velocities (generated through a random number seed) to check the structural convergence. First, analysis of structure and dynamics was done using the crystal structure as reference for three different types of structural data subsets within each state of the NADP/H-cofactor: the entire dimer, the monomers comprising dimer, and the domains (α-helical and Rossmann-type domains) within each monomer, in addition to dynamics during the simulations (see Figure 1 for cofactor-bound crystal structure of YqhD).

#### 2.1.1. YqhD Dimer

During the simulations, the populated dimer conformations have an average root mean square deviation (RMSD) of 0.28 ± 0.05 nm and 0.24 ± 0.04 nm when bound with NADP and NADPH cofactors, respectively. Radius of gyration (Rg) of dimer for the crystal structure is 2.71 nm. During the simulations, the YqhD dimer has conformations with an average 2.75 ± 0.01 nm Rg, which is slightly less compact than the crystal structure and is consistent with solution phase dynamical sampling. The cofactor binding and protonation states affect the dimer compactness during the simulations resulting in a less compact structure with NADPH and NADP cofactors than with NADPH(OH)_2_.

#### 2.1.2. YqhD Domain

A Rossmann-type domain is involved in NADP cofactor binding. With NADP-bound, the domain shows an average RMSD of 0.17 ± 0.03 nm with slightly higher variations than NADPH-bound (average RMSD of 0.15 ± 0.02 nm). The α-helical domain is a metal-binding domain and has an average RMSD of 0.11 ± 0.02 nm for NADP-bound and 0.13 ± 0.03 nm for NADPH-bound YqhD. Monomers bound to reduced cofactor show slightly higher deviations than monomers bound to oxidized cofactor. Both domains within each monomer become slightly more compact in the simulations with Rg values of 1.57 ± 0.01 for Rossmann-type and 1.63 ± 0.01 for α-helical domains during the simulations, compared to 1.60 nm for Rossmann-type and 1.65 nm for α-helical domains in the crystal structure.

#### 2.1.3. YqhD Monomer

**Figure 2:**
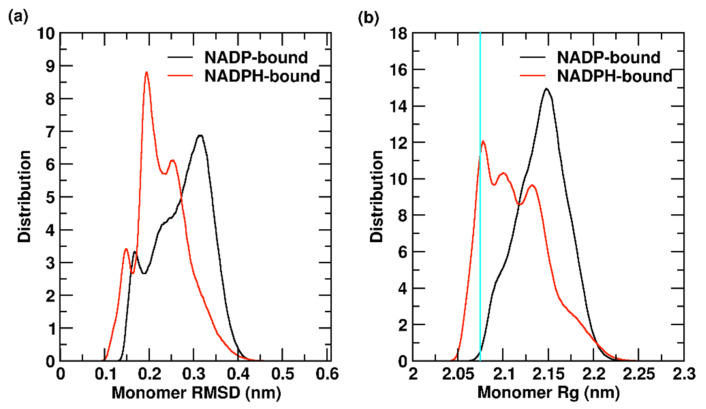
The distribution of backbone (**a**) root mean square deviation (RMSD) and (**b**) radius of gyration (Rg) values are shown for the YqhD monomers. The vertical, cyan-colored bar shows the Rg value observed in the crystal structure.

When comparing monomers in the 2 µs aggregated trajectory, it can be seen that the oxidation state of the NADP/H cofactor affects the overall monomer conformation significantly. NADP-bound monomer shows an average backbone RMSD of 0.27 ± 0.06 nm with slightly higher variations than NADPH-bound monomer, which has an average RMSD of 0.23 ± 0.05 nm. The distribution of backbone RMSD and Rg values for YqhD monomers is reported in Figure 2. YqhD monomers bound to NADP show higher deviation from the reference crystal structure. The RMSD distribution ranges from 0.13–0.45 nm, with a main peak at 0.31 nm and two additional peaks at 0.23 and 0.17 nm. NADPH-bound YqhD has a higher probability of remaining closer to the reference structure, showing a main peak at 0.19 nm and two additional sharp peaks at 0.25 and 0.15 nm; however, the RMSD distribution is broader, ranging from 0.09–0.47 nm. The Rg value of monomer is 2.07 nm in the crystal structure. NADP-bound monomers show an average Rg of 2.14 ± 0.02 nm, while NADPH-bound monomers have an average Rg of 2.11 ± 0.03 nm. For NADPH-bound monomers, Rg values show a broad distribution ranges from 2.04–2.27 nm with three peaks at 2.07, 2.10 and 2.13 nm. NADP-bound monomer exhibits a narrower Rg distribution, ranging from 2.04–2.25 nm, with a main peak at 2.15 nm and a wider shoulder at 2.09 nm. Overall, YqhD monomer bound with reduced NADPH cofactor populates conformations (two peaks) with lower RMSD (<0.2 nm) and Rg (<2.1 nm) values, i.e., close to the crystal structure that has the modified NADPH(OH)_2_ cofactor. Thus, some of the structural differences in YqhD monomer relative to the crystal structure can be attributed to the changes in the oxidation states of the cofactor (reflecting relevant, functional states of NADP/H bound to the enzyme); the molecular details are discussed later.

It is worthwhile to keep in mind that the RMSD and Rg values are calculated using the crystal structure as a reference, which may not reflect the functional enzyme structure due to its modified cofactor (NADPH(OH)_2_). Still, the crystal structure gives a good starting point to witness how the enzyme samples different conformations during the binding of oxidized and reduced cofactors. In the simulations, YqhD shows slightly higher structural flexibility, populating diverse conformations with a more compact yet broader, distinctive distribution of Rg values when bound to NADPH (required for reductase function) compared to NADP (released for enzyme turnover). The population of conformations towards lower RMSD and Rg values in the simulations with reduced cofactor indicates the slight change in protein conformation in NADPH binding relative to NADP binding.

**Figure 3:**
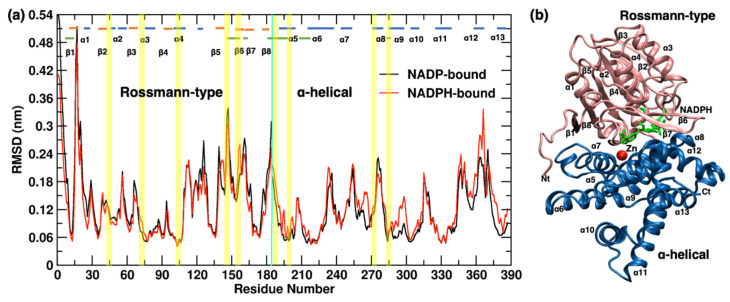
(**a**) Backbone RMSD per residue for the domains of monomers bound to NADP (black) and NADPH (red) with respect to the crystal structure. Horizontal bars show α-helices (blue), β-sheets (orange), and the residues involved in dimer and monomer interactions (cyan). The cofactor-binding region in Rossmann-type domain and metal-binding regions within the α-helical domain are shown in yellow vertical bars. (**b**) YqhD monomer is in cartoon representation with bound NADPH (green licorice representation) and Zn^2+^ (red sphere) in the active site. Helices, beta sheets, and C and N terminals are labeled.

Figure 3a shows the per-residue backbone RMSD for the YqhD domains illustrated in Figure 3b. High deviations are restricted mainly to loop regions and the N- and C- terminus. Both the monomers show significant deviations in the loops that are present at the dimer interface (between monomers) and the interface between domains within each monomer (marked by the cyan colored horizontal bar in Figure 3a). Monomers bound to NADPH show a higher deviation in loop regions α6/α7, α7/α8, α9/α10 and α12/α13 of the metal-binding helical domain then the NADP-bound monomer.

### 2.2. Hydrogen Bonding in YqhD Enzyme

Inter-monomer and interdomain hydrogen bonds were calculated in the crystal structure and during the simulations of YqhD bound to NADP/H. Hydrogen bonds were calculated using a distance between acceptor and hydrogen donor of 3.5 Å and an angle of <30° among acceptor, donor, and hydrogen as criteria. The dimer has ~8 inter-monomer hydrogen bonds, and monomers A and D have ~7 interdomain hydrogen bonds during the simulations (averaging over data collected every 10 ps). The hydrogen bonds discussed below are present for a cumulative total of >30% of the time points analyzed in the trajectories.

#### 2.2.1. Inter-Monomer Hydrogen Bonding

During the simulations, eight unique inter-monomer hydrogen bonds were observed in the dimer bound to NADP/H involving residues of Nt, β1, α5/α6, α6, and α7. The crystal structure also has seven unique hydrogen bonds between monomers, involving the residue pairs Leu1-Tyr238/Asp239 (Nt- α7), Asn3-Lys16 (Nt- β1), Phe4-Phe14 (β1- β1), Asn5-Arg11 (β1- β1), Leu6-Ile12 (β1- β1), Asp209-Asn243 (α5/α6- α7), and Lys211-Glu226/Asp227/Asn243 (α6- α6, α7). During the simulations, these hydrogen bonds were also observed in YqhD bound to NADP/H involving the residue pairs: Leu1-Tyr238 (Nt- α7), Asn3-Lys16 (β1- β1), Phe4-Phe14 (β1- β1), Asn5-Arg11 (β1- β1), Leu6-Ile12 (β1- β1), Asp209-Asn243 (α5/α6- α7), Lys211-Asp227/Asn243 (α6- α6/α7), and Arg215-Glu226 (α6- α6). NADP/H binding results in a dimer conformation that is less compact than the crystal structure with a slightly higher Rg value, but it maintains the hydrogen bonds observed in the crystal structure.

#### 2.2.2. Interdomain Hydrogen Bonding

Seven interdomain hydrogen bonds were observed in the crystal structure of monomers bound to NADPH(OH)_2_ involving the residue pairs Asn2-Gly259 (Nt- α7/α8), Lys16-238Tyr (β1/α1- α7), Asn145-Thr249 (β5/β6- α7/α8), Thr157-Lys359 (β6/β7- α12), Asp159-Lys359/His363 (β7- α12, α12/α13), Thr180-Arg241 (β8/α5- α7) and Pro184-Val188 (β8/α5- α5). During the simulations, five interdomain hydrogen bonds (with both NADP, NADPH bound) involve the residues of Nt [Leu1-Asn208/Asp209 (α5/α6) and Asn2-Gly259 (α7/α8)], β1 [Thr8-Asn253 (α7)], and β8/α5 [Thr180-Arg241 (α7), Pro184-Gln187/Val188 (α5)]. In addition, other hydrogen bonds involving residue pairs Thr142-Asp194 and Asp176-Tyr238 were observed in NADP-bound monomer. On average, three interdomain hydrogen bonds observed in the crystal structure between Asn2-Gly259, Thr180-Arg241 and Pro184-Val188 occurred frequently during the simulations. Observed changes in the hydrogen bonding interactions evidence conformational changes in the monomer to accommodate NADP/H cofactors.

### 2.3. Cluster Analysis of YqhD Enzyme

**Figure 4:**
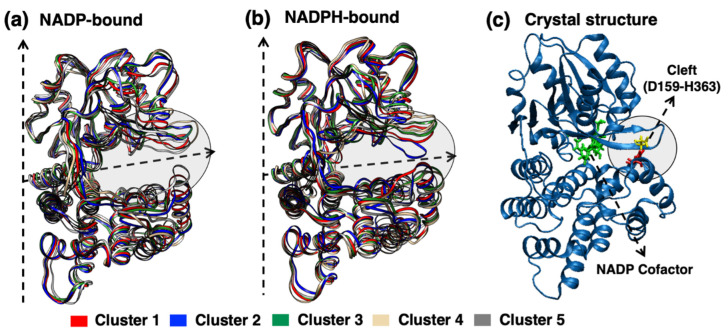
The representative conformations of the first five clusters (colored by red, blue, green, tan, and gray color, respectively) of homodimer (**a**) NADP-bound and (**b**) NADPH-bound are superimposed and represented as ribbons. The dotted arrows show the monomer- and domain- interface. The region involved in the opening/closing of the interdomain cleft is highlighted. (**c**) Interdomain cleft residues D159 and H363 are represented in the NADPH-bound monomer of crystallographic structure. Cluster 1 (in red color) shows partially closed conformations in both NADP/H bound- Yqhd monomer, while Cluster 2 (in blue color) is open confirmation in NADP-bound and closed in NADPH-bound monomer.

Conformational differences observed due to the cofactor oxidation/reduction state were further quantified using cluster analysis on monomers (see Section 4.4 for details). In the combined trajectory, a total of 54 (NADP) and 43 (NADPH) clusters were obtained for the monomer using an RMSD cutoff of 0.13 nm. Figure 4 represents the first three clusters of NADP-bound and NADPH-bound monomers in red, blue and green colored ribbons, respectively. These first three clusters comprise 62.7% (30.8%, 19.3%, and 12.6%) and 72.9% (46.0%, 13.8%, and 13.1%) of the total monomer conformations occupied by YqhD for NADP- and NADPH-bound enzyme, respectively.

**Figure 5:**
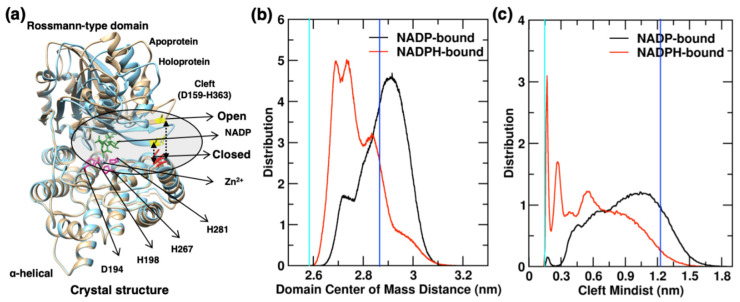
(**a**) Crystallographic structure of the YqhD monomer as apoprotein and holoprotein is in tan and sky-blue colored cartoon representation, respectively, with bound NADP (green licorice representation) and Zn^2+^ (violent sphere) in the active site [7]. Cleft residues and Zn^2+^ binding residues are labeled. (**b**) The distribution of distances between Rossmann-type and α-helical domains is shown using the center of mass. (**c**) The distribution for cleft minimum distances using residue pair D159-H363 is shown for NADP- and NADPH-bound monomers during the simulations. The cleft distances observed in the apo- and holo- YqhD protein crystal structures are marked with blue (apo) and cyan (holo) bars.

By looking at the superimposed mean cluster structures and crystal structure illustrated in Figure 4, it can be seen that the primary differences in monomer conformations arise from the position of the β6/β7 loop region (circled) of the Rossmann-type domain and the α8/α12 helix of α-helical domain. Figure 5a shows the superimposed crystallographic structures of apo- and holo- proteins. The β6/β7 loop region is in open conformation in the apo form of the YqhD crystal structure [7] (and closed in the holoenzyme), indicating that they comprise an interdomain cleft which opens and closes for cofactor binding and release. In this side-by-side comparison of the monomer, it can be seen that, with both cofactors, these loops are significantly dynamic. The highly populated conformations of NADPH-bound involve both monomers within the dimer remaining with a partially closed cleft (cluster 1), closed cleft (cluster 2), and open cleft (cluster 3). On the other hand, when oxidized NADP cofactor is bound, each monomer within the dimer samples more conformations with open cleft. All three of the highly populated conformations of NADP-bound enzyme are open cleft, indicating that cofactor oxidation state has an effect on the structures and populations of the open and closed conformation. Figure 5b represents the distribution of distances using the center of mass of two domains in the simulations as a global measure of the opening and closing of the domains. Domain distances observed in the crystal structures are shown in Figure 5b by vertical cyan and blue colored lines for the holo- and apo- enzymes, respectively. The NADPH-bound monomer remains in more closed and partially closed conformation, indicated by two main peaks for the distances between domains at 2.68 nm and 2.73 nm, respectively. A third peak is also observed at 2.83 nm, which is close to the one observed in the apoprotein, at 2.86 nm. In contrast, the NADP-bound monomer has the main peak at 2.90 nm for distances between domains, indicating a population of more open conformations, and a second small peak at 2.72 nm for sampling partially-closed structures; the molecular details are discussed later.

### 2.4. Interdomain Cleft

The cofactor binding site in each YqhD monomer spans the interdomain face. The residues of β6/β7 loop region act as the clamp at the mouth of the cofactor binding region, which remains in open conformation in the apo form [7] and closed in the holoenzyme crystal structures. The closed conformation of holoenzyme has hydrogen bond Asp159-Lys359/His363 involving residues D159 of β6/β7 loop region in the Rossmann-type domain and residues K359/H363 of α12 helix in the α-helical domain. For a better understanding of cleft opening/closing and its relationship with cofactor binding/release, we defined interdomain cleft using residue pair D159-H363 at the mouth of the cofactor binding site, on the interface of the Rossmann-type and α-helical domains (see Figure 5a). In the crystal structure, the holoenzyme has a distance of 0.16 nm for cleft, while the apo-enzyme has distances of 1.25 nm for cleft [7], which indicates the involvement of cleft opening/closing in cofactor binding and release. Figure 5a shows the residue pair D159-H363 which is involved in interdomain cleft formation and clamps the NADP/H cofactor in the binding site. The interdomain cleft shows hydrogen bonding between residue pairs D159-K359/H363 and K160-H271/E272 in the crystal structure and simulations. YqhD has ~5% D159-H363 hydrogen bond existence in the NADPH-bound monomer trajectory; however, its occurrence is negligible in NADP-bound monomers.

Figure 5c shows the distribution of cleft distances using the residue pair D159-H363. Cleft distances observed in the crystal structures are shown in Figure 5c by vertical cyan and blue colored lines for the holo- and apo-enzymes, respectively. NADP-bound monomer shows a wider distribution range of cleft distances, from 0.14–2.0 nm, compared to those in NADPH: 0.14–1.7 nm. It can be seen in Figure 5c that the distribution of cleft distances shifts to the right with oxidized cofactor, reflecting wider openings of the interdomain cleft in NADP-bound monomers. Monomers sample conformations with interdomain cleft distances using residue pairs D159-H363: (i) less than 0.55 nm, representing closed cleft as observed in holoenzyme, (ii) ranges from 0.55–1.0 nm, for partially-closed cleft representing transition states and (iii) more than 1.0 nm, having open cleft as observed in protein. NADPH-bound monomers have two peaks in the distance distribution (Figure 5c) at 0.16 and 0.28 nm for cleft distances representing closed confirmations, and the third peak at 0.58 nm showing partially-closed conformations. However, NADP-bound monomers show a peak at 0.17 and a broader distribution of cleft distances, encompassing partially-closed and, mainly, open cleft confirmations. Changes in interdomain cleft distances occurred mainly due to a major shift in the positions of helix α12 and beta sheets β6 and β7 (see the circled region in Figure 5a), but it is a cooperative motion (involving the cleft forming regions of both domains). Cleft distance data indicate that residue D159 of cleft works as the clamp to monitor cleft opening and closing for cofactor binding and release, and its movement is affected by cofactor oxidation state.

#### 2.4.1. Interdomain Opening-Closing Cleft Dynamics

**Figure 6:**
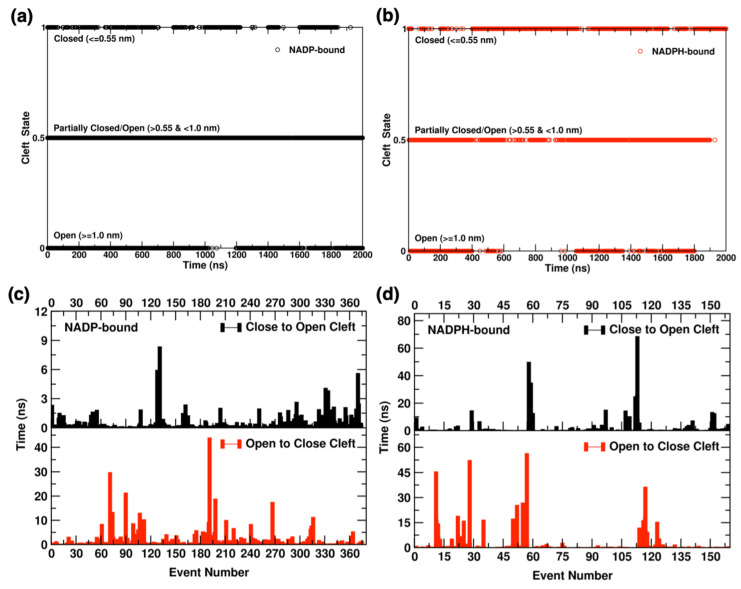
Open, partially closed/open, and closed states of the interdomain cleft observed using residue pair D159-H363 in YqhD monomer bound to (**a**) NADP and (**b**) NADPH cofactors during the simulations. Waiting time is for each instance of cleft opening and closing in YqhD monomer bound to (**c**) NADP and (**d**) NADPH.

To characterize the cleft dynamics, we evaluated the minimum distance data of cleft opening/closing residues D159 and H363. For Yqhd monomer, we set a distance of <0.55 nm for closed states and >1.0 nm for open states. By assigning the open, partially-closed, and closed states a value of 0, 0.5 and 1, respectively, we were able to generate a graph of state vs time, shown in Figure 6a,b. The state-time analysis is performed on the 2 µs long aggregated trajectory involving 10 sets of NADP/H-bound monomer data. Within the aggregated data, the NADP-bound monomer is in the open state for 41% of the time, partially closed 49%, and closed for 10%. In contrast, the NADPH-bound monomer highly populates the closed state (44% of time points) and exists in the transitional conformation 39% and open state for 17% of the time points in the aggregated trajectories. The state population data indicate an efficient sampling of protein conformations during the 10 independent sets of simulations. From the simulation data, the effect of cofactor oxidation state is clearly evident on the occurrence of one state over another (see Figure 6a,b). For example, the NADP-bound monomer is prone to access open states, while NADPH-bound monomer remains in closed states more frequently. We also evaluated cleft minimum distance data to calculate waiting times for the enzyme to remain in a conformation before switching cleft state (i.e., switching conformation from closed to open, and vice versa). Figure 6c,d shows the cleft dynamics in NADP/H-bound monomers as the number of events and wait time for each instance required for switching from closed to open state and vice versa. The equilibrated starting structure of YqhD homodimer is in the closed state in both NADP/H-bound monomer. During the simulations, the monomer rapidly fluctuates between open and closed conformations, resulting in waiting times ranging from picosecond to nanoseconds for switching of states. The interdomain cleft remains more dynamic in NADP-bound monomer with a total of 750 events of cleft opening/closing, compared to NADPH-bound domain with 318 events of state switching. The waiting time for state switching remains under 1 ns in 75% of cleft opening/closing events of NADP-bound monomers and 80% of NADPH bound monomers. For switching from closed to open state, the average wait time was 2.4 ns in NADPH-bound monomer and 409 ps in NADP-bound monomer. The longest wait time of switching from closed to open state was 8.3 ns in NADP-bound monomer and 68 ns in NADPH-bound monomer. However, switching from open to closed state occasionally required even longer average waiting times of 2.9 ns (maximum 56 ns) for NADPH-bound and of 1.4 ns (maximum 43 ns) for NADP-bound monomer. The cleft dynamics data evidences the effect of cofactor oxidation state on enzyme dynamics. Simulation results indicate that (a) the cleft dynamics are influenced by the cofactor oxidation state and (b) opening of the interdomain cleft facilitates the release of NADP cofactor (below), which is consistent with the experimentally observed properties of the biocatalysts such as the higher affinity of YqhD enzyme for NADPH cofactor indicated by its lower K_M_ value of 0.008 mM [11] over NADP cofactor, K_M_ = 0.150 mM [21].

#### 2.4.2. Cofactor Binding and Release in YqhD Enzyme

**Figure 7:**
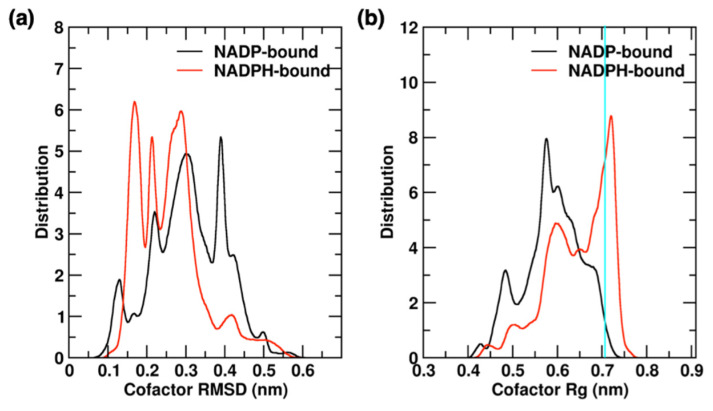
Distributions of (**a**) RMSD and (**b**) Rg values are shown for NADP and NADPH cofactors in the aggregated trajectories of monomers. Cyan colored horizontal bar represents cofactor Rg in the crystal structures.

The cofactor was found to be quite flexible during simulations, which can be quantified with variations in cofactor RMSD and Rg values. Figure 7 shows the distribution of RMSD and Rg values in the 2 µs of aggregated trajectory data, analyzing NADP/H-bound within each monomer of the YqhD dimer. The NADP cofactor shows a broader distribution of RMSD values, with five peaks at 0.40, 0.30, 0.21, 0.13 and 0.50 nm, compared to NADPH with four peaks at 0.15, 0.30, 0.22 and 0.42 nm. The RMSD distribution for NADP cofactor is shifted to the right, sampling conformations having higher RMSD values relative to the holoprotein crystal structure. Rg values of the NADP/H cofactors indicate its compactness during the simulations. The NADPH cofactor shows a main peak at 0.73 nm that is close to the Rg of cofactor observed in the crystal structure, representing an extended cofactor confirmation bound in the intradomain region of each monomer. Additional peaks are observed at 0.60 nm and small peaks at 0.44 and 0.49 nm. However, the Rg distribution of the NADP cofactor is shifted to the left (more compact), with three main peaks at 0.58, 0.60 and 0.49 nm. During the simulations, extended confirmations of cofactor were sampled more frequently by NADPH than NADP. This detail is in agreement with the previous observation that the NADP-bound monomers sample more open and partially closed conformations, having a looser cofactor binding, with the open cleft conformation facilitating cofactor release.

**Figure 8:**
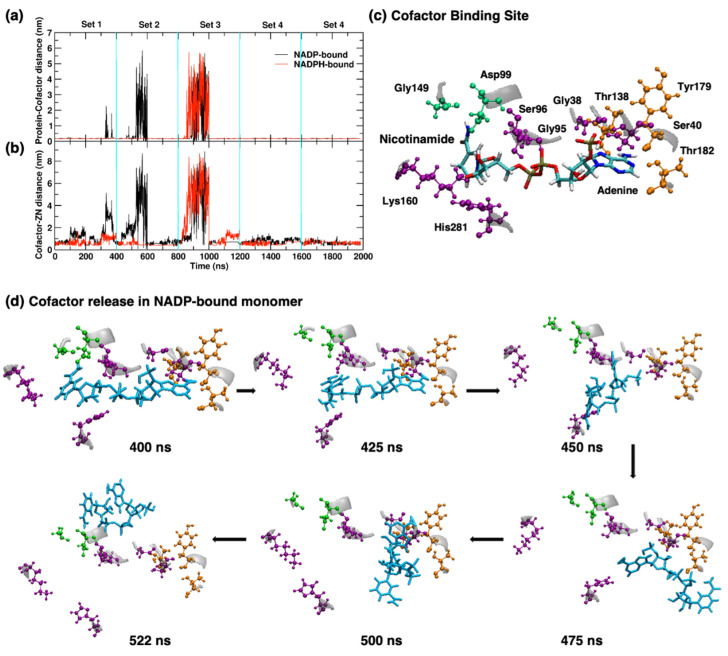
The minimum distance is in nm between (**a**) protein and cofactor, and (**b**) cofactor and zinc ion as a function of time in the aggregated trajectory of monomer bound to NADP and NADPH. Large distances correspond to cofactor release events. (**c**) Cofactor binding site shows residues forming hydrogen bonds in the starting structure of YqhD monomer. Cofactor binding residues are colored based on their hydrogen bonding partner, being adenine moiety in orange, nicotinamide in green color, and nucleotide or phosphate group in purple colors. (**d**) Snapshots of NADP-bound YqhD monomer showing cofactor binding site starting from the equilibrated structure in Set 2 trajectory followed by every 25 ns interval up to cofactor release event in the second set of YqhD simulation.

Subsequently, we observed an average minimum distance in the monomer trajectories between protein and cofactor, and cofactor and Zn^2+^ of 0.16 and 0.45 nm, respectively, to the crystal structure (see Figure 8a,b). The distances between cofactor and protein had significant differences during the simulations and are related to the binding of cofactor in each monomer. Zn^2+^ has an average distance of 0.25 nm from residues Asp194, His198, His267 and His281 during the simulations as observed in the crystal structure (see Figure 5a). A minimum of one water and Ala141 remain within 0.19 nm of the Zn^2+^ over the course of both NADP and NADPH simulations. Within 0.35 nm distances from Zn^2+^, an average of 12 ± 3 and 10 ± 3 water molecules were present in NADP and NADPH-bound YqhD monomer, respectively. A higher number of water molecules were present close to Zn^2+^ in open conformations than the closed one: 15 ± 2 water molecules for NADPH and 15 ± 7 for NADP-bound YqhD during the time-interval 800–1000 ns, which shows the population of open conformations indicated by higher distances between cofactor and protein in Figure 8a. 

NADP-bound YqhD showed cofactor release in three out of five sets of simulation trajectories, while NADPH-bound monomer exhibited cofactor release only once, in Set 3. The NADPH-bound monomer has a more rigid cofactor binding, which coincides with a conformational change in the cofactor binding pocket. The changes in the cofactor distances from the protein are related to the dynamic nature of cofactor in the binding site, as observed earlier with deviations in RMSD and Rg values of monomer and cofactor during the simulations. A total of 15 hydrogen bonds were observed in the starting structure of the cofactor binding site to the cofactor. Figure 8c shows the residues involved in hydrogen bonding with NADP/H cofactor. Hydrogen bonds involve the adenine moiety with residues Thr138, Tyr179, and Thr182; the dinucleotide moiety and phosphate groups with residues Gly38, Ser40, Gly95, Ser96, His281, and Lys160; and the nicotinamide moiety with residues Asp99 and Gly149. During the simulations, Lys160 has no hydrogen bond with NADP/H cofactor, while His281 shows hydrogen bond existence in 5% of NADP-bound monomer trajectory frames. Excluding these latter two hydrogen bonds present in the starting structure, NADP/H-bound structures have conserved hydrogen bonds involving the adenine moiety, nucleotide and phosphate group in 50% of the aggregated trajectory frames. The NADP-bound monomer has only one hydrogen bond involving Leu279 and nicotinamide moiety for 6% existence in the aggregated trajectory. However, NADPH bound monomer forms three potential hydrogen bonds involving Asp99, Ser144, and Gly149 in ~30% of the aggregated trajectory. In both NADP/H-bound structures, hydrogen bonds involving the adenine moiety were observed more frequently (50% time points) than with nicotinamide moiety (6% in NADP and 30% in NADPH-bound monomers), which also supports our earlier observations of lower Rg and indicates the population of a bent conformation of NADP/H cofactor in open cleft states before release shown in Figure 8d. Overall, these results indicate that cofactor release is associated with an increase in the interdomain cleft (>0.55 nm), followed by loosened cofactor binding at cleft distances of 0.55–1.0 nm and loss of hydrogen bonds involving nicotinamide moiety, a change in cofactor conformation to a more compact structure (lower Rg), and, finally, release.

## 3. Discussion and Conclusions

Molecular dynamics simulations were performed on the YqhD dimer, with oxidized and reduced NADP/H cofactors bound in aqueous solution. The starting point of the simulations came from the only solved crystal structure of holo YqhD [7], which has a modified NADPH(OH)_2_ cofactor, and Zn^2+^ in the active site of only one of the monomers within the functional dimer unit. Simulations were run on the functional structure of YqhD, with Zn^2+^ present in each monomer, and with NADP^+^ and NADPH cofactors, using careful methodology to prepare these structures (i.e., repairing the crystal structure artifacts). Good conformational sampling of YqhD bound to NADP/H was obtained by performing five sets of simulations that were assigned different initial velocities from a Maxwell-Boltzmann velocity distribution at 300 K. The structures remain conserved throughout the simulations, regardless of the oxidation state of NADP/H cofactor. So, we compiled a combined trajectory of the 200 ns from the set of five simulations with both NADP/H cofactors and obtained sampling of various conformations representing opening and closing of the cofactor-binding interdomain cleft. Each monomer of the YqhD dimer showed coordinated cleft opening/closing, with both oxidized and reduced NADP/H, via the movement of β6/β7 and α12 regions in the interdomain cleft. The cleft remains open in the apo form of the YqhD crystal structure [7], indicating that it opens and closes for cofactor binding. Observations of cofactor release during MD simulations confirm the movement to an open-cleft conformation prior to the release of both NADP and NADPH. The sampling of open-cleft conformations depends on the cofactor oxidation state. NADP-bound monomers tend to sample more open conformations, with cleft distances ranging from ~0.7–1.8 nm (cf. ~0.7–1.5 for NADPH, 1.25 nm for apoprotein crystal structure [7]).

The dynamics of cleft opening/closing were also found to depend on the oxidation/reduction of the cofactor. With both cofactors, YqhD undergoes periods of rapid cleft opening and closing, sampling open conformations similar to the apoenzyme [7]. NADP-bound monomer scarcely populates conformations with a closed cleft in 10% of time points and remains in a partially closed state for 49% of time points or adopts open cleft conformation in 41% of time points. NADPH-bound monomers remain in closed conformation for 44% and spend only 17% in open state conformations. The maximum waiting time of transition from closed to open state was observed to be ~8 ns in NADP-bound monomer and 68 ns in NADPH-bound monomer. However, NADP-bound monomer underwent a total of 750 transitions between open and closed states, compared to 318 total cleft state transitions observed for NADPH-bound monomer, indicating a higher propensity for cleft opening dynamics when the enzyme is bound to the oxidized cofactor. Out of five independent simulations, cofactor release was observed in three sets of NADP-bound and only one set of NADPH-bound structures. This suggests an entropic, dynamics-based preference for cofactor release when NADP is bound.

Significant differences were observed even in the conformation of NADP/H cofactor, concomitant with conformational differences in the monomers. The differences in cofactor-enzyme interactions and YqhD conformational dynamics may rationalize differences in the Michaelis constant, K_M_, which depends on cofactor binding (k_f_) and release (k_r_) substrate-binding rates as K_M_ = (k_r_ + k_cat_)/k_f_. The higher number of cofactor-monomer hydrogen bonds between NADPH-bound vs. NADP-bound YqhD may lead to higher binding rates and/or slower release rates for NADPH, resulting in the higher affinity for NADPH indicated by its lower K_M_ value (0.008 mM [11] vs. 0.150 mM [21] for NADP). Furthermore, the less frequent sampling of open conformations with NADPH-bound YqhD may hinder NADPH release, dropping k_r_ and subsequently K_M_ for NADPH. Meanwhile, conformational dynamics appear to promote the more facile release of NADP, following hydride transfer from NADPH to aldehydes. Thus, the conformational differences induced by cofactor oxidation state, dynamical effects of more-frequent cofactor cleft opening with NADP, and differences in hydrogen bond motifs may lead to preferential kinetics for the aldehyde reductase activity of YqhD. These findings raise questions about whether enzymes with higher alcohol dehydrogenase activity show a dynamic preference for the release of reduced cofactor. Results of this study enhance our basic understanding toward this class of enzyme, with the possible application of guiding the rational design of YqhD to enhance substrate affinity and biocatalyst efficiency.

## 4. Materials and Methods 

### 4.1. Starting Coordinates

The crystal structure of YqhD (PDB ID: 1OJ7, 2.0 Å resolution) [7] was used to extract the starting coordinates for setting up MD simulations. The starting coordinates include a dimer (crystallographic monomers A and D, shown in Figure 1a) with bound Zn^2+^, modified NADP cofactor as NADPH(OH)_2_ due to oxidative stress, and crystallographic waters. In the crystal structure, only one monomer (D) of the YqhD homodimer has Zn^2+^ in the active site. Herein, monomer D is used to model the starting structure of YqhD homodimer with bound Zn^2+^ and NADPH(OH)_2_ cofactor using PyMOL molecular graphics system [33]. Monomer D was duplicated and superimposed onto monomer A using the root mean square deviation (RMSD) minimization criteria of PyMOL to generate a homodimer structure with two bound Zn^2+^ ions for MD simulations.

### 4.2. Modeling of NADP/H Cofactor

In the crystal structure of YqhD homodimer, [7] NADP cofactor is present as NADPH(OH)_2_ (see Figure 1b), with modification at the C5 and C6 positions of the nicotinamide moiety. The native oxidized cofactor (NADP) was modeled by removing the hydroxyl moieties at the fifth and sixth positions in the nicotinamide ring. The reduced cofactor (NADPH) was prepared by modifying the oxidation state of the C4 atom of the nicotinamide ring. Forcefield parameters come from CHARMM 36 force field [34] for Zn^2+^, NADP, and NADPH cofactors. Throughout this paper, NADP/H is used to signify the NADPH cofactor generally, whether in its oxidized (NADP^+^) or reduced (NADPH) state. When a specific oxidation state is indicated, NADP and NADPH are specified.

### 4.3. Molecular Dynamics Simulations

The CHARMM 36 force field [34] was used for the simulations summarized in Table 1 using the GROMACS software package version 5.1.3 [35]. The YqhD dimer bound to Zn^2+^ and NADP/H cofactors was centered in a cubic periodic box (~11 nm^3^) and set to have a distance larger than 1 nm from any side of the box. Solvent molecules having any atom within 0.15 nm from the protein were removed. TIP3P model [36,37] was used for explicit water solvent. Sodium counter ions were added by replacing the solvent molecules at the sites of most negative electrostatic potential to provide the box with a total charge of zero. The protonation state of residues was assumed to be the same as that of the isolated amino acids in solution at pH 7. The LINCS [38] algorithm was used to constrain all bond lengths and the SETTLE [39] algorithm was used for the water molecules. Electrostatic interactions were calculated using the Particle Mesh Ewald method [40]. For the calculation of long-range interactions, a grid spacing of 0.12 nm combined with a fourth-order B-spline interpolation was used to compute the potential and forces between grid points. A non-bonded pair-list cutoff of 1.4 nm was used and updated at every five time-steps. V-rescale thermostat [41] was used to keep the temperature at 300 K through a weak coupling of the system to an external thermal bath with a relaxation time constant τ = 0.1 ps. The pressure of the system was kept at 1 bar using Berendsen’s barostat [42] with a time constant of 1 ps. A time step of 2 fs was used to integrate the equations of motion.

First, the simulated systems were energy minimized, using the steepest descent algorithm, for at least 5000 steps to remove clashes between atoms that were too close. After energy minimization, all atoms were given an initial velocity obtained from a Maxwell-Boltzmann velocity distribution at 300 K to start the MD simulations. The system was initially equilibrated by 30 ps with position restraints on the heavy atoms of the dimer to allow relaxation of the solvent molecules. After the equilibration procedure, position restraints were removed, and the system was gradually heated from 50 K to 300 K during 200 ps of simulation. The equilibrated structure was used to perform MD simulations in the NPT ensemble for 20 ns at 300 K. The final conformation of the 20 ns isothermal-isobaric NPT ensemble was used to set up five sets of 200 ns independent simulations in the canonical NVT ensemble initiating with five different velocities.

The starting coordinates were adopted from the crystal structure which has modified cofactor (NADPH(OH)_2_); hence, the equilibrated conformations of YqhD bound to NADP and NADPH cofactors were obtained after a 20 ns NPT simulation, which was used as the starting point for the final production run of 200 ns long NVT simulations. The starting crystallographic coordinates of the YqhD homodimer were used as the reference structure for the analysis of the trajectories to characterize the conformational changes induced by a change in the oxidation state of NADP/H cofactor. The analysis was focused on three sets/sub-structures within the simulation data, considering dimer, monomer, and domain in a 1 µs aggregated trajectory for each cofactor oxidation state (NADP and NADPH). Structural analysis includes the root mean square deviation (RMSD), root mean square fluctuation (RMSF), radius of gyration (Rg), and cofactor binding interactions (Zn^+2^ and NADP/H), comparing these with respect to the crystal structure.

### 4.4. Cluster Analysis

The conformational diversity of the structures generated during the MD simulations was characterized using the Gromos [43] clustering algorithm. In this method, an RMSD cutoff criterion is used to assign a structure in a cluster based on the root-mean-square differences of selected atoms among the conformations obtained from the simulations. An RMSD cutoff of 0.13 nm was used to determine neighboring backbone atom conformations of YqhD monomers to discriminate among the less varying conformations. For the cluster analysis, a total of 52,815 structures were evaluated for each monomer within the dimer over the 2 µs aggregated trajectory, using time intervals of 100 ps. [33]

## Figures and Tables

**Table 1:** Simulation summary for the YqhD homodimer in aqueous solution.

Enzyme	Cofactor	Atoms	Water Molecules	Counter Ions	No. of Simulations	Time (ns)
YqhD Dimer	NADP	128,728	40,363	14 Na^+^	5	200
YqhD Dimer	NADPH	128,723	40,360	16 Na^+^	5	200

## Data Availability

The data presented in this study are available on request from the corresponding author.
